# High-Sensitivity Differential Sensor for Characterizing Complex Permittivity of Liquids Based on LC Resonators

**DOI:** 10.3390/s24154877

**Published:** 2024-07-27

**Authors:** Zhongjun Li, Shuang Tian, Jiaxin Tang, Weichao Yang, Tao Hong, Huacheng Zhu

**Affiliations:** 1 School of Electronic Information Engineering, China West Normal University, Nanchong 637002, China; zhongjunli@stu.cwnu.edu.cn (Z.L.); tianshuang2024@stu.cwnu.edu.cn (S.T.); jiaxintang2022@stu.cwnu.edu.cn (J.T.); taohong@cwnu.edu.cn (T.H.); 2 College of Electronics and Information Engineering, Sichuan University, Chengdu 610064, China

**Keywords:** differential sensing, stepped impedance, LC resonators, microstrip planar sensor, complex permittivity

## Abstract

This paper proposes a high-sensitivity microstrip differential sensor for measuring the complex permittivity of liquids. The prototype of the differential sensor was formed by cascading two LC resonators on a microstrip transmission line based on stepped impedance. A strong electric field was found to be distributed in the circular patch of the LC resonator; therefore, a cylindrical micropore was set in the center of the circular LC resonator to measure the dielectric sample, which maximized the disturbance of the dielectric sample on the sensor. By optimizing the size of the circular LC resonator, a high-sensitivity sensor circuit was designed and manufactured. The complex permittivity of the test sample was calculated by measuring the transmission coefficient of different molar concentrations of ethanol–water solutions. The experimental results show that the designed differential sensor can accurately measure the complex permittivity of liquid materials with an average sensitivity of 0.76%.

## 1. Introduction

Complex permittivity is an important parameter that describes the electrical characteristics of materials under an applied electromagnetic field, and microwave measurement technology is a commonly used method for detecting complex permittivity [1,2,3,4,5,6], which can be further applied in gas detection [7], solid medium characterization [8], crack detection [9], oil sample detection [10], and biomedical applications [11]. Depending on the different design structures of sensors, microwave sensors can be classified into resonant and non-resonant types. The non-resonant method characterizes samples through changes in transmission and reflection coefficients within a wide frequency band, which has strict requirements for sample preparation [12]. On the other hand, resonant sensors can provide more accurate measurement results at a single or discrete frequency point. Therefore, microwave sensors based on resonant methods are more important in the detection of solid and liquid materials.

Waveguide cavities are commonly used devices in the design of microwave sensors, which detect samples by placing them in the region where the electric field is most concentrated in the cavity. However, they tend to be in a huge volume, which is not conducive to portability [13]. In order to reduce the volume of the sensor, planar circuits, such as substrate-integrated waveguides (SIWs) and microstrip lines, have received widespread attention in the design of microwave sensors. The SIW structure reduces the volume and cost of the sensor while maintaining the advantages of traditional waveguides. The design of the SIW sensor is usually based on the perturbation theory, establishing micropores in the region where the electric field is highly concentrated to maximize the interaction between the material to be measured and the electric field, thereby obtaining the maximum sensitivity [14,15]. In addition, etching structures such as interdigital structures [16], split-ring resonator (SRR) structures [17,18], complementary split-ring resonator (CSRR) structures [19], and stepped-impedance resonator structures [20] in the microstrip circuit can enhance the constraint on the electric field and achieve the detection of permittivity. In particular, microfluidic sensors have attracted much attention in the design of sensors due to their small required test sample volume, which can reduce the waste of test samples. On the one hand, microfluidic sensors can be achieved by establishing micropores in the region where the electric field of the sensor is highly concentrated [14,15,17]; on the other hand, microfluidic sensor design can be achieved by building liquid channels on resonant structures.

Sensitivity is one of the important indicators in sensor design, and a high-sensitivity sensor is conducive to identifying slight changes in the dielectric constants of materials to be tested. In [15], a microfluidic sensor based on an SIW was proposed, which achieved high sensitivity by creating micropores in the area where the electric field was highly concentrated. In [21], a microfluidic sensor based on the CSRR structure was proposed, realizing a highly sensitive sensor by creating microfluidic channels on both sides of the CSRR. In [22], an ultra-high sensitivity microfluidic sensor for the measurement of the dielectric constant of an ethanol–water solution was designed based on a stepped-impedance resonator. However, as the sensitivity of the sensors increased, the cross-sensitivity caused by environmental factors such as temperature and humidity also increased, which led to measurement errors. 

Microwave differential sensors are an effective solution to improve robustness, capable of suppressing unwanted environmental influences [23]. Differential sensors feature two sensor elements of the same size, and, due to their structural symmetry, the two resonant frequency points of the sensor perfectly overlap. During measurement, one sensor element is used as a reference element, while the other is used as a measuring element for placing the material to be tested [24]. The presence of the material to be tested interferes with the field distribution of the resonator used as the measuring element, causing a shift in the resonant frequency, resulting in two resonant frequencies. Therefore, the difference between the measurement values of the two resonant elements can be used as the output variable of the sensor to improve robustness to changes in environmental factors. In [18], a sensor designed by using two microstrip lines loaded with an SRR was proposed. However, this sensor required four ports, which was not conducive to observation and measurement. To avoid this drawback, an interesting solution is to fabricate two single-port devices and use the difference in reflection coefficients between the two ports as the output variable. This approach satisfies the need for differential measurement while reducing the number of ports and improving measurement convenience. For example, in [6], a differential sensor for measuring the permittivity of glycerol–water solutions was designed by fabricating two identical single-port RLC resonators on a dielectric substrate for differential measurement. Additionally, differential sensors with resonant devices loaded on a single transmission line can also be specifically designed. For instance, in [25], an interesting differential sensor for solid material measurement was designed by loading a pair of stepped-impedance resonators. In [12], a differential microwave sensor for characterizing the permittivity of liquids was designed by serially connecting two identical complementary SRRs on a microstrip line. By loading the sample, the reflection coefficient response at the measurement port changed accordingly, while the reference response remained unaffected. Therefore, the design focus of differential sensors lies in eliminating the coupling between resonant devices to prevent coupling effects from impacting sensor performance.

This article proposes a simple differential LC sensor based on a microstrip stepped-impedance structure for characterizing the complex permittivity of liquids. An LC resonator was designed by creating a micropore in the circular area, with a highly concentrated electric field of the stepped-impedance resonator. Then, two identical LC resonators were cascaded at half-wavelength intervals on a microstrip transmission line to form a microwave differential sensor. In Section 2, the basic structure and equivalent circuit of the sensor are presented, and the equivalent circuit is used to explain the working principle of the sensor. A circuit model of a highly sensitive microfluidic sensor for measuring the complex permittivity of liquids was obtained through the simulation and optimization of the proposed sensor structure. Section 3 describes the verification of the performance of measuring the complex permittivity by measuring ethanol–water solutions with different water molar concentrations, and the average sensitivity of the sensor is calculated and compared with other sensors.

## 2. Sensor Structure, Operation, and Design

### 2.1. Sensor Structure

**Figure 1:**
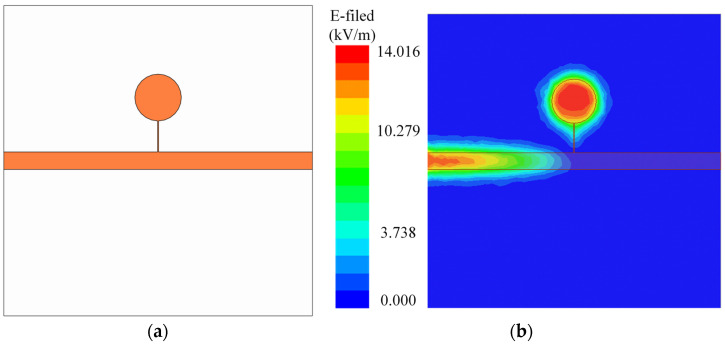
(**a**) Structural diagram of stepped-impedance resonator. (**b**) Electric field distribution.

The proposed stepped-impedance resonator was composed of a microstrip transmission line connected to a high-impedance transmission line and a low-impedance circular patch, as shown in Figure 1a. The electric field distribution on the metal ground plane of the sensor was obtained using eigenmode simulation in ANSYS HFSS 18.2, ANSYS, America, as shown in Figure 1b. 

**Figure 2:**
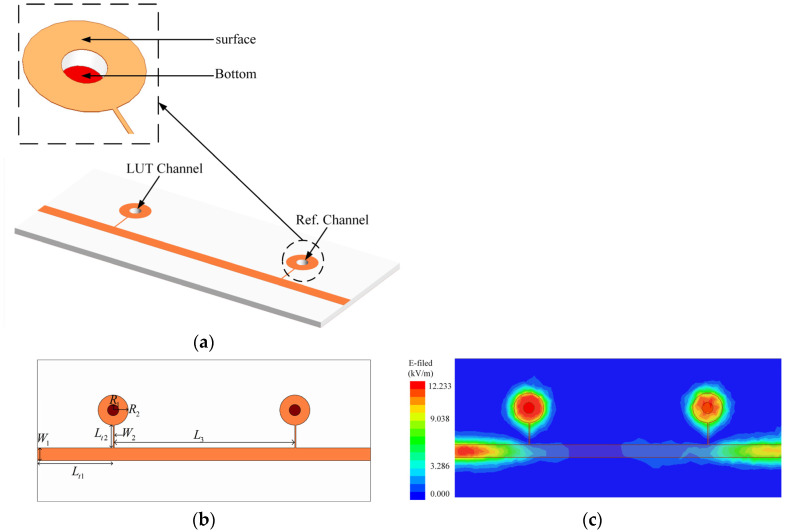
Differential sensor structure diagram: (**a**) three-dimensional view; (**b**) vertical view; (**c**) electric field distribution.

From the electric field distribution results of the resonator in Figure 1b, it can be seen that the electric field was highly concentrated in the low-impedance circular patch region. Therefore, a micro-hole was created at the center of the low-impedance circular region, forming an LC resonator, as shown in Figure 2a. The hole was created by chipping off the top copper and dielectric substrate, and the bottom of the hole was sealed with copper. Utilizing this region for the dielectric constant detection of samples allowed the maximum electric field to pass through the micro-hole loaded with the liquid under testing (LUT), enhancing interaction with the electric field and providing the highest sensitivity.

Robustness is an important indicator in sensor design, and differential sensing measurement is a powerful solution to improve the robustness of sensors. Therefore, a pair of identical LC resonators was cascaded on the microstrip transmission line to form differential sensing measurements. Compared with a single LC resonator, this scheme improved the anti-interference ability of the sensor. However, when the distance between the resonant elements of the differential sensor was too close, coupling could occur between the resonant elements. To avoid the impact of such coupling and improve the performance of the differential sensor, according to [25], resonators can be placed at positions separated by half a wavelength on the main transmission line to eliminate the coupling between resonant elements. Since the input impedance of the load seen through the half-wavelength line is unchanged, the structure behaves as if the LC resonators are physically located at the same junction (due to the periodicity, this stays at an integer multiple of half wavelength). Therefore, this study used a differential sensor composed of two identical LC resonators, with two resonant devices placed at positions separated by half a wavelength on the microstrip main transmission line to eliminate the impact of coupling on the differential sensor.

The circuit prototype of the differential sensor proposed in this article is shown in Figure 2. The upper surface of the sensor was composed of a microstrip transmission line cascading a pair of stepped-impedance LC resonators, and micro-holes were created in the two circular low-impedance areas to place the LUT. The bottom surface of the sensor was a layer of copper with a thickness of 0.035 mm, serving as the grounding surface. Due to the presence of two sensing elements in the differential sensor, one of the stepped-impedance LC resonators could be used as a reference element during the testing process, while the other stepped-impedance LC resonator was used to place the LUT. When unloaded, the resonant frequencies of the two resonant elements completely overlapped due to structural symmetry. The electric field distribution on the metal ground plane of the sensor was concentrated in the low-impedance region, as shown in Figure 2c. After introducing fluid into the micro-hole of the resonant element used for measurement, its resonant frequency shifted. Therefore, the frequency shift and peak attenuation of the resonant frequency of the measurement element relative to the unloaded element could be used to characterize different dielectric samples.

### 2.2. Operating Principle

**Figure 3:**
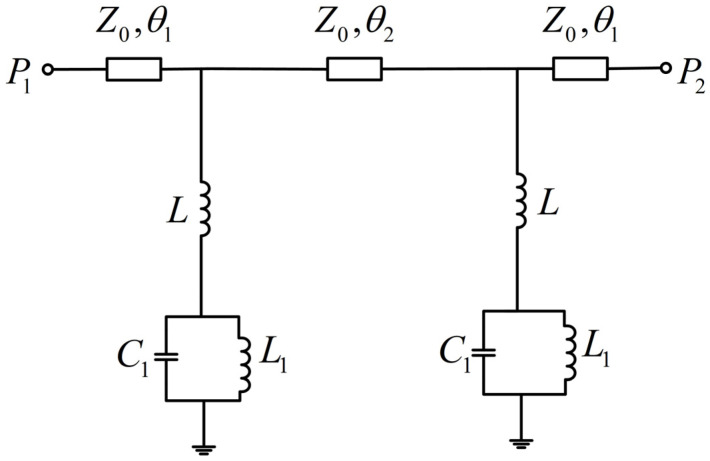
Equivalent circuit model.

The working principle of the LC resonator based on the stepped-impedance structure can be explained by perturbation theory [15]. According to the theory of dielectric perturbation, introducing a dielectric sample to the region where the electric field is highly concentrated will disturb the electric field. The unique dielectric properties and losses of the sample will cause the resonant frequency shift of the sensor and the quality factor change. By creating a micro-hole in the center of the low-impedance region, the liquid to be tested can be introduced. In this case, the high-impedance segment of the stepped-impedance resonator is represented by inductance *L*, the low-impedance region is represented by inductance *L*_1_ and capacitance *C*_1_ in parallel [26,27], and the perturbation caused by the dielectric sample is represented by capacitance *C*_2_. Based on the prototype of the sensor, an equivalent circuit model was obtained, as shown in Figure 3.

According to the equivalent circuit of the circuit, the resonant frequency of the circuit was as follows:

(1) $$f=\frac{1}{2\pi }\sqrt{\frac{L+L_{1}}{LL_{1}C_{1}}}$$

**Figure 4:**
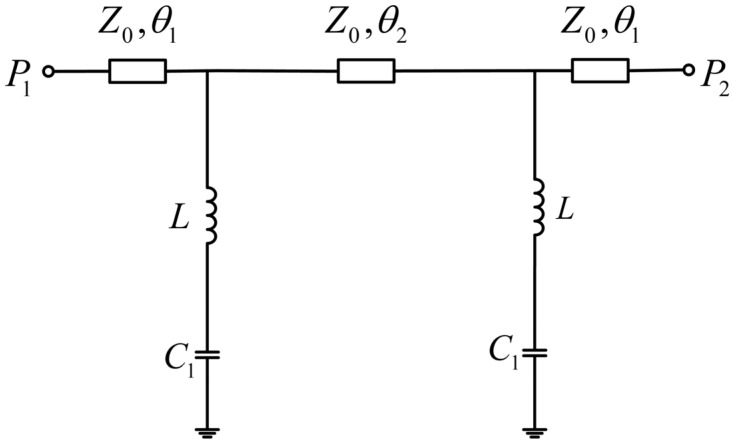
Simplified equivalent circuit model.

It can be seen that the unloaded resonant frequencies of the LC resonators serving as the reference element and the measurement element overlapped. After the LUT was loaded, the added capacitance *C*_2_ affected the resonant frequency. In this case, *C*_1_ in the resonant frequency expression was modified to (*C*_1_ + *C*_2_) so that the resonant frequency was reduced, while the resonant frequency of the LC resonator serving as the reference element remained unchanged. This resulted in two resonant frequency points in the circuit, enabling differential sensing measurement. The equivalent circuit in Figure 3 included the case where the metal ring exhibited higher impedance after a micro-hole with a radius of *R*_1_ was created in the low-impedance region. However, when the metal ring exhibited low impedance, the low-impedance area could be represented by capacitor *C*_1_, while the inductance *L*_1_ could be ignored [26]. The simplified equivalent circuit is shown in Figure 4.

At this point, the resonant frequency of the circuit could be simplified as follows:

(2) $$f=\frac{1}{2\pi \sqrt{LC_{1}}}$$

### 2.3. Design of the Sensor

**Figure 5:**
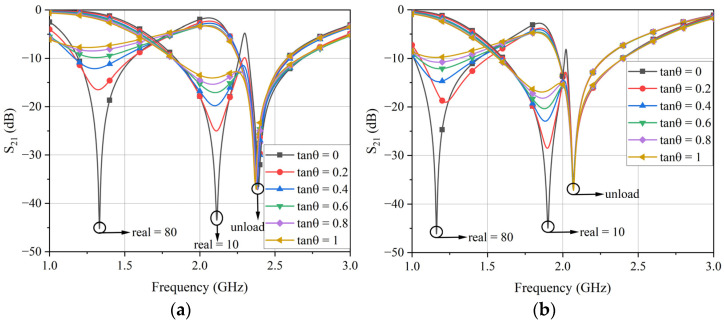

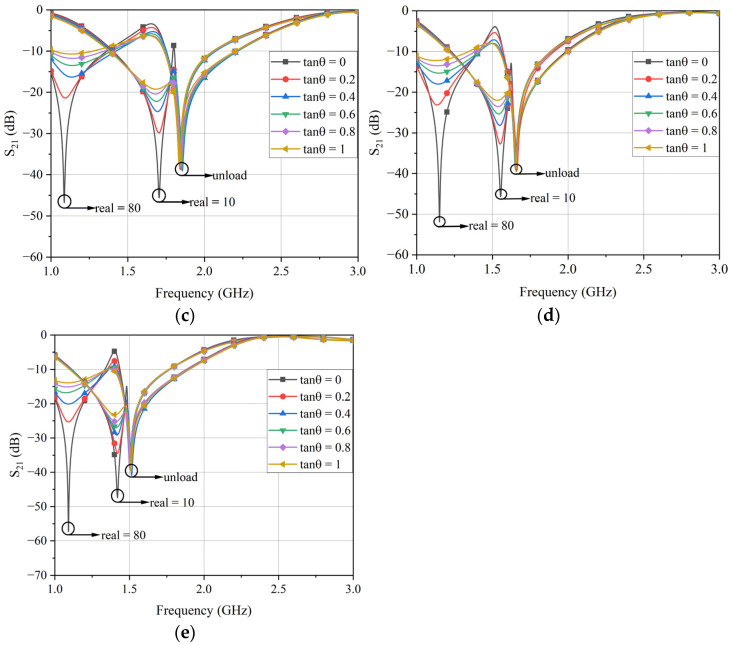
The transmission coefficient response of sensors with different *R*_2_ values: (**a**) *R*_2_ = 3 mm; (**b**) *R*_2_ = 3.5 mm; (**c**) *R*_2_ = 4 mm; (**d**) *R*_2_ = 4.5 mm; (**e**) *R*_2_ = 5 mm. tanθ is the loss tangent.

Since the electric field of the sensor was mainly concentrated in the circular LC resonant region, in order to achieve the optimal design of the differential sensor, in this section, the size of the circular LC resonant region will be discussed, and its response to different dielectric samples will be analyzed. In the process of simulation analysis, the full-wave electromagnetic simulation software ANSYS HFSS, ANSYS 18.2, America was used. The frequency range was randomly set as 1–3 GHz and the center frequency was set as 2 GHz. The substrate used for the sensor design was Rogers 4003c board with a thickness of 1.524 mm, which has a relative permittivity of 3.55 and a loss tangent of 0.0027. In order to achieve microfluidic sensor measurement and reduce the waste of test samples, the radius *R*_1_ of the measurement hole was set to 1.5 mm. The microstrip transmission line was designed with 50 ohms as the standard to achieve impedance matching. The width *W*_1_ of the microstrip transmission line was determined to be 3.37 mm based on the designed center frequency. At the same time, for the sake of achieving a high impedance of stepped impedance, the microstrip line width *W*_2_ and length *L*_t2_ were set to 0.2 mm and 6 mm, respectively. When analyzing the response of the change in the radius *R*_2_ of the circular LC resonant region to the change in the permittivity, the variation range of *R*_2_ was set to 3–5 mm and the step interval was set to 0.5 mm. In addition, to achieve a relatively wide permittivity measurement, samples with relative permittivity of 10, 30, 50, and 80 were used for simulation analysis. Since the actual test samples could have losses, to measure samples with larger losses, the loss tangent of the samples was set to 0–1 with an interval of 0.2 during the simulation analysis, and the transmission response of the differential sensor was obtained. Due to the similarity of the transmission response, only the simulation results with relative permittivity of 10 and 80 are shown in Figure 5.

**Figure 6:**
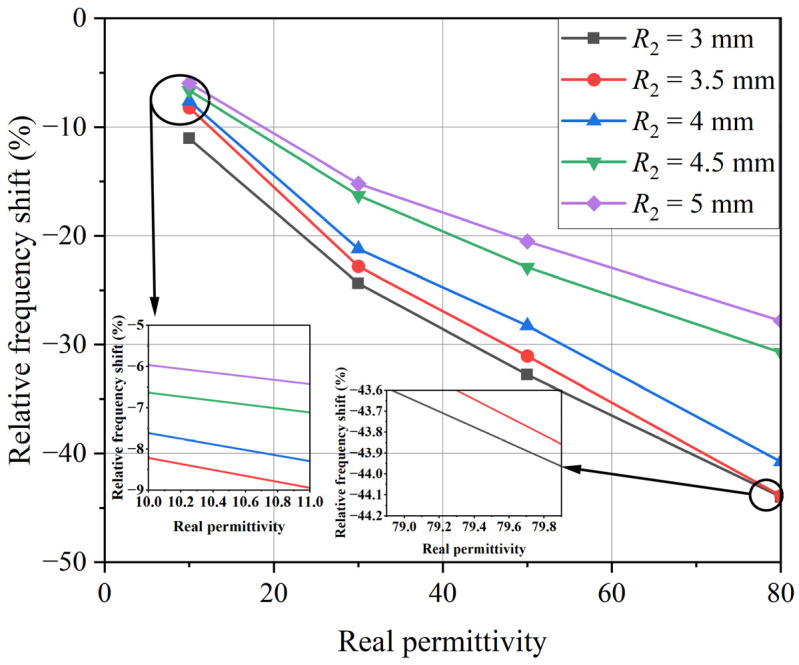
Simulated relative frequency shift as a function of the relative permittivity of the LUT with different *R*_2_ values.

Through simulation, it was found that adjusting the radius *R*_2_ of the circular low-impedance region could change the resonant characteristics of the device, thus achieving the optimization effect of the device. As *R*_2_ increased, the resonant frequency of the sensor decreased, and the frequency offset between the loaded resonant frequency and the unloaded resonant frequency also decreased. The relative frequency was determined by the frequency shift introduced by the LUT, which was defined as the ratio of the resonant frequency shift to the unloaded resonant frequency [15]. The relative frequency shift was used to evaluate the performance of the sensor, and the relative frequency offset without loss under different *R*_2_ sizes was calculated, as shown in Figure 6.

As can be seen from the simulation results in Figure 6, when *R*_2_ decreased, the interaction between the LUT and the electric field became stronger, enhancing the sensitivity of the sensor. However, the quality factor of the loaded sensor decreased as the loss tangent of LUT increased. In particular, the quality factor became very small for *R*_2_ = 3.0 mm. Therefore, the peak became inconspicuous, which could lead to measurement accuracy. With the increase in the size of *R*_2_, the detection capability of the sensor’s transmission response for the sample’s loss tangent also improved. Considering both the relative bandwidth for permittivity measurement and the measurement range of the loss angle, the optimized *R*_2_ was set to 4 mm, with a corresponding separation of 48 mm between the two resonators. The resonant frequency of the sensor without load was 1.833 GHz. It is worth noting that the area ratio of the unloaded micro-hole to the low-impedance region was about 14% according to the final size of the sensor, and the inductance *L*_1_ of the metal ring could be ignored. Therefore, according to the methods reported in the literature [26], the capacitance *C*_1_ and inductance *L* in Figure 4 could be calculated using Advanced Design System 2020, which and were 5.29 pF and 1.43 nH, respectively.

### 2.4. Influence of the Test Liquid Shape

**Figure 7:**
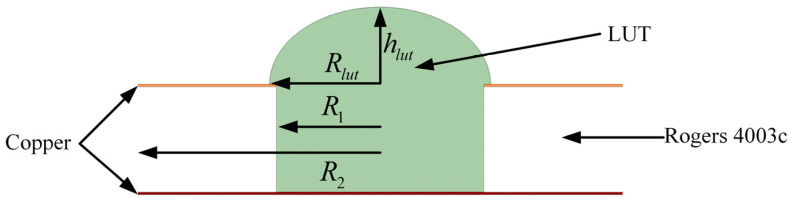
Cross-section of LUT.

In addition, the properties of ethanol–water solution cannot be fully simulated by simulation software ANSYS HFSS, ANSYS 18.2, which may lead to differences between simulation and measurement data. In order to reduce this difference, more liquid is often introduced in the actual measurement [14]. However, the introduction of too much liquid will cause the droplets to overflow the measuring hole, as shown in Figure 7.

**Figure 8:**
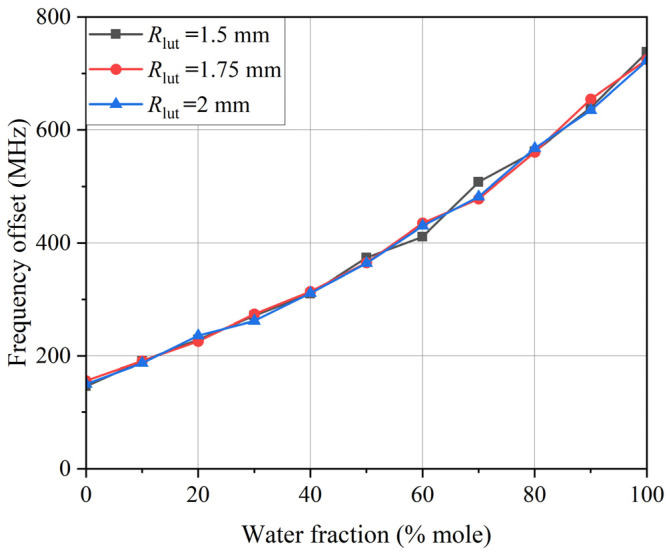
Frequency offset of the proposed sensor to LUT with different *R*_lut_.

Therefore, the influence of droplets with the same volume but different radius *R*_lut_ and height *h*_lut_ on the sensor performance was simulated. In the simulation process, *R*_lut_ was set to 1.5 mm, 1.75 mm, and 2 mm, respectively, and the corresponding *h*_lut_ was 1.27 mm, 0.94 mm, and 0.715 mm, respectively. The simulation results using ethanol–water solutions are shown in Figure 8. According to the simulation results, when the measured volume remained the same, the shape of the droplet had little effect on the change of the resonant frequency of the sensor.

## 3. Measurement and Results

### 3.1. Sensor Calibration

**Figure 9:**
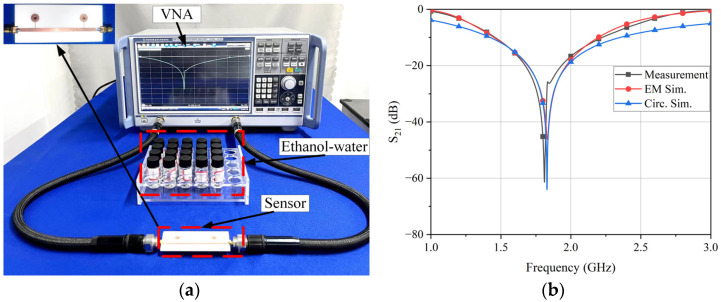
(**a**) Sensor measurement system. (**b**) Measurement and simulation of unloaded transmission response.

The proposed differential sensor based on stepped impedance was manufactured and tested. Two subminiature version A (SMA) connectors were welded to the two ends of the microstrip transmission line of the sensor. An experimental system was set up with a vector network analyzer (ZNB 40, Rohde & Schwarz, Munich, Germany) and the differential sensor, as shown in Figure 9a. First, the transmission coefficient S_21_ of the sensor under unload was measured, and the unloaded transmission response of simulation and measurement is shown in Figure 9b. It can be seen from the measurement results that the unload transmission response of the differential sensor was 1.811 GHz, which was basically consistent with the result of 1.833 GHz from the circuit model and electromagnetic simulation. The slight frequency offset may have been caused by the tolerance of circuit processing.

The characteristics of ethanol–water mixed solutions have been studied in detail in the previous literature [28]. Ethanol and water solutions and their mixtures cover a relatively wide range of permittivity values, which were further adopted as test samples in this study to investigate the sensor’s performance, as shown in Figure 9a. To achieve more accurate measurement results from the sensor, calibration was required. Calibration samples with a water molar concentration ranging from 0% to 100% were prepared using a mixture of ethanol and deionized water, with a 10% interval between each sample’s molar concentrations. The complex permittivity of these calibration samples at 20 °C and 1.811 GHz is shown in Table 1 [28].

**Figure 10:**
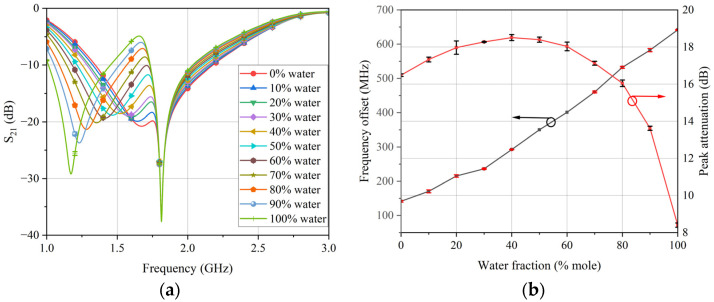
(**a**) Transmission coefficient response of calibration samples. (**b**) Frequency shift and peak attenuation of calibration samples.

Compared to other methods, BP neural networks are capable of fitting more complex relationships and producing more accurate results [29,30,31,32,33]. Therefore, this study adopted a BP neural network to map the relationship between the peak attenuation, frequency shift of the sensor, and the real and imaginary parts of the complex permittivity. At an ambient temperature of 20 °C, 17 μL of each calibration sample was dropped into one of the measurement holes using a microliter pipette, and the transmission coefficient response was recorded using the vector network analyzer. In order to verify the stability of the sensor, three groups of experiments were conducted and error bars were plotted for the measurement results, as shown in Figure 10a. Based on the results in Figure 10a, the frequency shift and peak attenuation of the calibration samples were extracted, as shown in Figure 10b. It is worth noting that the peak attenuation was defined as the difference between the peak of the loaded resonant frequency and the peak of the unloaded resonant frequency. It can be seen from the test results that the transmission frequency response of the sensor changed as the dielectric properties of the calibration samples varied. The frequency shift from the unloaded sample to water was 643 MHz, covering the change in the real part of the permittivity from 1 to 79. The results of multiple measurements of the sensor’s transmission response were stable.

**Figure 11:**
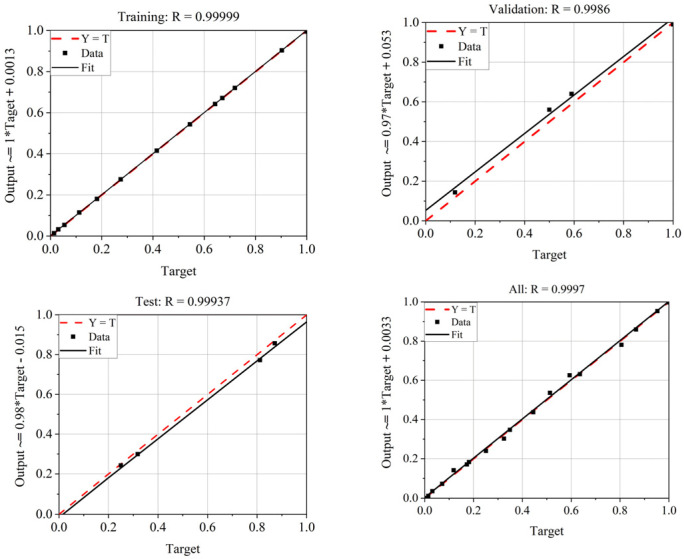
Neural network goodness of fit.

Then, the frequency shift and peak attenuation of the calibration samples were used as the input of the neural network model, while the real and imaginary parts of the corresponding complex permittivity of the calibration samples were set as the output, thus establishing a dual-input and dual-output BP neural network. Goodness of fit is often used to evaluate neural network models, and a value close to 1 indicates that the model fits the data well. After training, the goodness of fit of the BP neural network model was obtained, as shown in Figure 11. Training, Validation, Test, and All in Figure 11 represent the data correlation relationships for the training set, validation set, test set, and overall results, respectively. The regression value R represents the correlation between the predicted output and the target output, and the closer the R value is to 1, the closer the relationship between the predicted and output data is. It can be seen from the goodness of fit results that all indicators were very close to 1, indicating that the neural network model achieved a satisfactory fit between the frequency shift, peak attenuation, and the real and imaginary parts of the complex permittivity.

### 3.2. Sensor Performance Validation

**Figure 12:**
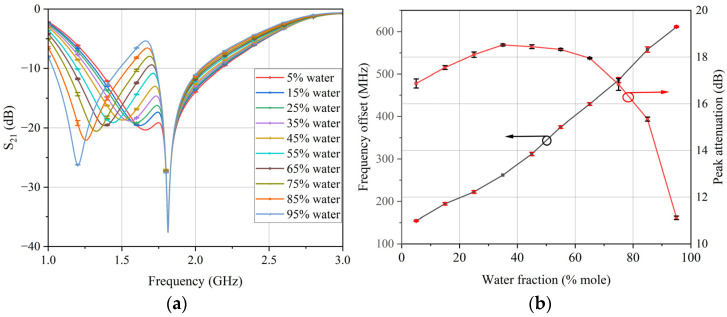
(**a**) Transmission coefficient response of test samples. (**b**) Frequency shift and peak attenuation of test samples.

Next, the proposed microstrip differential sensor was tested as a microfluidic sensor. In order to verify the accuracy of the designed sensor and BP neural network model in measuring permittivity, ethanol–water mixed solutions with a water molar concentration ranging from 5% to 95% with a 10% interval were introduced into the liquid channel for testing. At the same time, three groups of experiments were conducted to verify the reliability of the measurement results. In each test, the volume of the test sample used was 17 μL, and the ambient temperature was controlled at 20 °C. The actual transmission response measured by the sensor is shown in Figure 12a. The frequency shift and peak attenuation calculated based on the transmission response for different test samples are shown in Figure 12b. 

**Figure 13:**
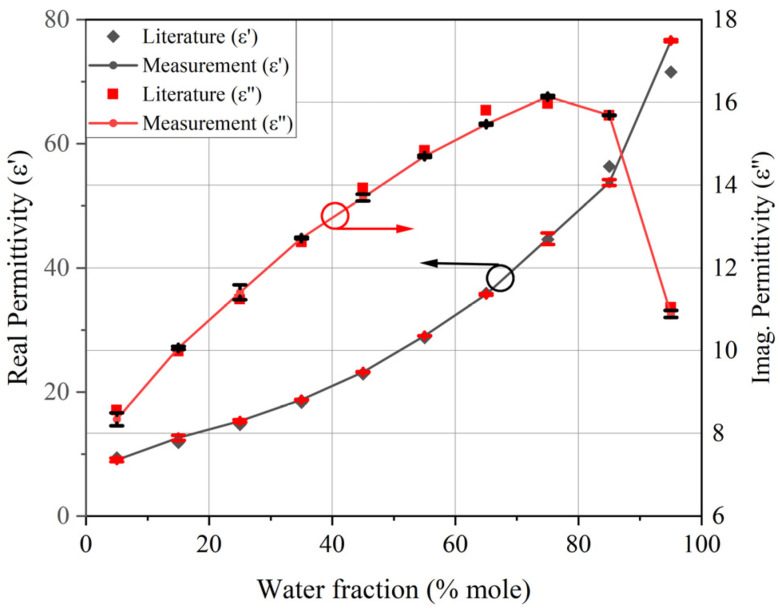
Comparison of complex dielectric constant values measured by the sensor and reference [28].

It can be concluded from the test results that the frequency response of the sensor varied with the dielectric properties of the test samples. Finally, the frequency shift and peak attenuation values of the test samples were input into the trained BP neural network model to calculate the real and imaginary parts of the complex permittivity. A comparison of the error bar results calculated based on three groups of experiments with the reference values reported in the literature [28] is presented in Figure 13.

From the measured results, it can be observed that compared to the reference values reported in the literature [28], the maximum error of the real part of the calculated complex permittivity was 7.09%, with an average error of 3.2%. For the imaginary part, the maximum error was 2.48% and the average error was 1.3%. From the measurement results, it can be seen that there was a certain error between the measured values and the literature reference values [28]. Since there were some fluctuations in the concentrations of the configured solutions, this may have introduced measurement errors. In addition, the fitting results of the neural network were different from the reference values. The superposition of these errors caused a deviation between the measurement results and the reference values, but the experimental results indicate that the designed sensor is capable of accurately measuring the complex permittivity of ethanol–water mixed solutions.

Table 2 lists the relevant data of various sensors reported in the literature, which were used to evaluate the performance of the proposed sensor. The sensitivity of the sensor was defined as [15]

(3) $$S=\frac{f_{r}-f_{0}}{f_{0}(\varepsilon^{\prime }-1)}$$

where *f*_0_ represents the reference resonant frequency of the sensor, *f_r_* represents the loaded resonant frequency of the sensor, and $\varepsilon^{\prime }$ represents the relative permittivity of the test sample. Based on the relationship (3) and the measured data, the sensitivity of the proposed sensor to ethanol–water solutions was determined. For the purpose of comparison, the average sensitivity *S*_av_ of the sensor was calculated according to the sensitivity of different samples. By comparing with other sensors, it could be observed that the proposed sensor provides higher sensitivity. In addition, the proposed sensor is a differential microfluidic sensor that has strong resistance to environmental interference and can reduce the waste of test volume. The sensor proposed in this paper can successfully detect the change in complex permittivity of ethanol–water solutions with differential functions and high sensitivity, thus showing strong competitiveness in the field of permittivity measurement.

## 4. Conclusions

This paper presented a microwave differential sensor composed of two LC resonators based on stepped-impedance structures for the measurement of microfluidic dielectric constants. The designed sensor enhanced the interaction between the dielectric material and the electric field by performing measurements in regions where the electric field was highly concentrated, enabling the microwave differential sensor to achieve high sensitivity. Subsequently, a BP neural network was utilized to establish a relationship between the frequency shift, peak attenuation of the dielectric sample, and the real and imaginary parts of the complex permittivity. Finally, the proposed sensor was used to measure the complex permittivity of ethanol–water mixed solutions. The experimental results demonstrate that the designed sensor exhibits good precision and sensitivity in measuring complex permittivity. The average errors of the real and imaginary parts of the complex permittivity were 3.2% and 1.3%, respectively, and the average sensor sensitivity was 0.76%. In summary, the sensor circuit designed in this study is simple in form, which is conducive to processing and manufacturing. In addition, the designed sensor has a differential measurement function, which improves robustness to environmental interference compared with ordinary sensors. Finally, compared with the other sensors listed in Table 2, it has the advantage of high sensitivity.

## Figures and Tables

**Table 1:** Complex permittivity of ethanol–water mixture at 1.811 GHz.

Water Molar Fractions/%	$\varepsilon^{\prime }$	$\varepsilon^{\prime \prime }$
0	8.12	7.92
10	10.49	9.32
20	13.08	10.70
30	16.46	12.00
40	20.33	13.09
50	25.89	14.52
60	31.49	15.04
70	39.55	15.69
80	50.01	16.08
90	63.11	14.08
100	79.57	7.83

**Table 2:** Comparison of various sensors.

Ref.	Type of Liquid	*f*_0_ (GHz)	$\varepsilon^{\prime }$ Range	*S*_av_ (%)	Differential
[6]	Glycerol–water	2.3	8.22–79.5	0.389	Yes
[12]	Ethanol–water	1.618	4–78	0.626	Yes
[14]	Ethanol–water	2.61	8–77.2	0.215	No
[15]	Ethanol–water	3.98	5.46–67.2	0.345	No
[17]	Ethanol–water	2.364	9–79	0.049	No
[19]	Ethanol–water	2.44	9.02–77.3	0.78	No
[21]	Ethanol–water	2	7.55–80.8	0.318	No
[22]	Ethanol–water	1.91	10.47–78	0.578	No
[23]	Urine–water	1.25	66–74	0.279	Yes
[24]	Isopropanol–water	1.05	8.4–80	0.059	Yes
This work	Ethanol–water	1.811	8.12–79.57	0.76	Yes

## Data Availability

Data are available upon request.
